# The Effect of Antimicrobial Photodynamic Therapy on the Healing of the Post-Extraction Socket of the Mandibular Third Molar: A Randomized Clinical Study

**DOI:** 10.3390/jcm14145029

**Published:** 2025-07-16

**Authors:** Alessia Pardo, Maria Lonardi, Annarita Signoriello, Gianluca Colapinto, Funda Goker, Margherita Tumedei, Massimo Albanese, Massimo Del Fabbro

**Affiliations:** 1 Dentistry and Maxillofacial Surgery Unit, Department of Surgery, Dentistry, Paediatrics and Gynaecology (DIPSCOMI), University of Verona, Piazzale L.A, Scuro 10, 37134 Verona, Italy; alessia.pardo@univr.it (A.P.); maria.lonardi@univr.it (M.L.); annarita.signoriello@univr.it (A.S.); massimo.albanese@univr.it (M.A.); 2 Department of Biomedical, Surgical and Dental Sciences, Università degli Studi di Milano, 20122 Milan, Italy; margherita.tumedei@unimi.it (M.T.); massimo.delfabbro@unimi.it (M.D.F.); 3 Department of Oral and Maxillofacial Surgery, Faculty of Dentistry, Istanbul Aydın University, 34295 Istanbul, Turkey; 4 Fondazione IRCCS Ca’ Granda Ospedale Maggiore Policlinico, 20122 Milan, Italy

**Keywords:** antimicrobial photodynamic therapy, mandibular molar, third molar surgery, tooth extraction

## Abstract

**Objectives**: We aimed to evaluate the efficacy of antimicrobial photodynamic therapy (aPDT) in promoting wound healing after the surgical removal of inferior third molars. **Methods**: Patients in need of unilateral mandibular third molar extraction were randomly assigned to either a test or control group before surgery. During the test, a photoactive substance activated with laser light (20 mW, 660 nm) was applied to the post-extraction site for 60 s before suturing to promote healing and disinfection. The control group did not receive any laser applications after tooth removal. The probing pocket depth (PPD), bleeding on probing (BOP), plaque index (PI), gingival recession (REC), and levels of clinical attachment loss (CAL) before surgery (T0), 14 days after surgery (T1), and after 3 months after surgery (T2) were evaluated for the adjacent second molar. Post-operative swelling, pain (VAS index), the number of painkillers taken, alveolar probing, and Landry’s healing index were recorded at T1. **Results**: Sixty-five patients, aged between 14 and 39 years, were assigned randomly to test (n = 32) or control (n = 33) groups. Five dropouts occurred. Post-operative swelling and the VAS index were significantly lower in the test group compared to the control (*p* = 0.002 and *p* = 0.04, respectively). All periodontal indexes except recession significantly worsened at T1 in both groups. After three months, a significant improvement for PPD, CAL, and PI was recorded in the test group compared to the control (*p* = 0.001). **Conclusions**: According to the results of this study, the use of aPDT seems to have a beneficial effect on post-operative swelling and pain, as well as the plaque index, in the short-term follow-up.

## 1. Introduction

The extraction of lower third molars is one of the most frequent procedures in oral surgery [1,2]. Unlike other teeth, third molars are often impacted, with a slightly higher prevalence in females than in males. For this reason, the extraction of these teeth generally requires a surgical approach involving the elevation of a mucoperiosteal flap and osteotomy to allow the use of elevators and the removal of the tooth either in pieces or as a whole, depending on the situation [1,2]. As a result, it is a more invasive procedure than simple dental extraction, which leads to a more challenging healing period for the patient, with complications such as pain, swelling, and trismus [2,3]. Studies in the literature suggest that the peak of pain reported by patients occurs 3–5 h after the local anesthetic wears off while swelling reaches its maximum in the first 24–48 h before gradually decreasing [4,5].

In some cases, post-extraction complications can be severe and can have a great impact on the patient’s quality of life. Like other routine surgical procedures, guidelines have been established for third molar extraction to help and lead professionals in various clinical scenarios to avoid unnecessary interventions that can cause complications. In the first few hours following the surgery, in addition to symptom onset, reparative mechanisms begin, contributing to the healing of the post-extraction site [4,5]. In some cases, patients also need additional grafting procedures [6]. Independent of the use of bone grafts, antimicrobial photodynamic therapy (aPDT) has been introduced to enhance healing and the disinfection of the extraction site [5,6]. aPDT uses a non-thermal photochemical reaction, which promotes the excitation of a nontoxic dye (photosensitizer) via light at an appropriate wavelength. This causes interactions with molecular oxygen and acts by damaging biomolecules selectively and destroying bacterial membranes [7]. The efficacy of this therapy in reducing bacterial load has been demonstrated in the literature, and it has been widely used in patients with periodontitis or peri-implantitis for several years [8]. Although the primary use of antimicrobial photodynamic therapy seems to be related to periodontal and peri-implant diseases, its use in oral surgery to disinfect the socket and reduce the risk of complications related to bacterial contamination of the surgical site should not be underestimated [9]. Furthermore, the biostimulator effect of the laser can promote tissue healing after surgery through vasodilation, the activation of microcirculation, and the enhancement of tissue metabolism, thus reducing the recovery time for the patient [10].

There are still a few papers in the literature that evaluate the effect of aPDT on post-operative healing after wisdom tooth extractions [7,11]. This study aimed to investigate the effect of aPDT on the healing of soft and hard tissues and post-surgical discomfort in subjects undergoing mandibular third molar extraction. The hypothesis is that photodynamic therapy accelerates and improves the healing process of soft tissues evaluated using periodontal indexes, especially in the first two weeks. The null hypothesis is that aPDT has no beneficial effects compared to spontaneous healing.

## 2. Materials and Methods

### 2.1. Study Type

This was a controlled, randomized, clinical and radiographic prospective study. The study was conducted in accordance with the ethical standards outlined in the 1964 Declaration of Helsinki and was approved by the Territorial Verona Ethics Committee (CESC VR-RO: Prog. 4242CESC). This report follows the CONSORT 2010 checklist for randomized trials [12]. The study was registered at ClinicalTrial.gov with the clinical trial number NCT06964178.

### 2.2. Inclusion Criteria

Healthy patients (ASA I/II according to the American Society of Anesthesiologists classification) requiring at least one mandibular third molar extraction, without preoperative significant pain or edema were included in the study after signing written informed consent; patients were aged between 14 and 39 years.

### 2.3. Exclusion Criteria

Patients with systemic diseases interfering with normal healing processes, those who were pregnant or lactating, patients with third molar buds, and patients with continuity between the roots of the lower third molar and the cortical bone of the mandibular nerve canal were excluded from the study.

### 2.4. Sample Size Calculation

**Figure 1:**
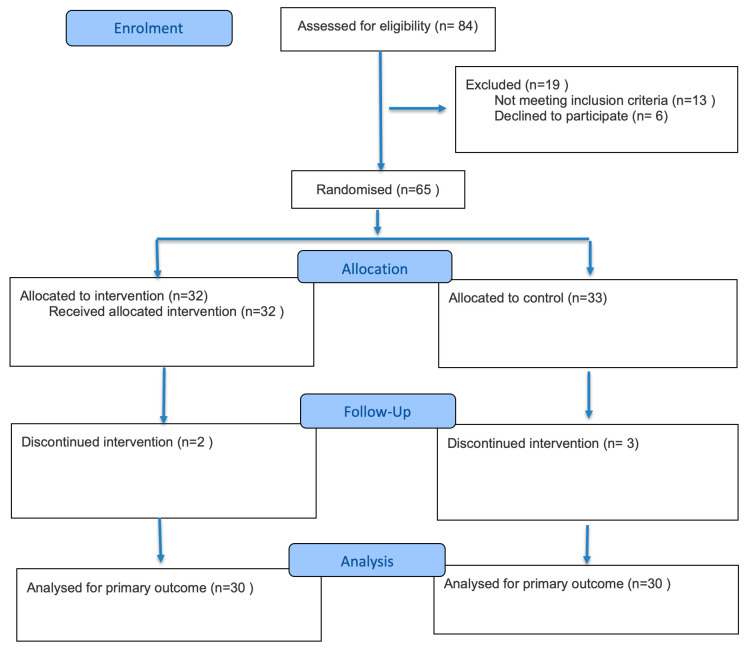
Consort diagram showing the phases of this randomized trial.

The sample size was estimated, assuming a 20% greater improvement for the probing pocket depth (PPD) measured in adjacent molar three months after surgery in the a-PDT group with respect to the control, with a standard deviation of 0.8 mm, a power of 80%, a bilateral confidence interval of 95%, and a 1:1 ratio between the two groups. The calculation was performed using Open-Epi software (Open-Source Epidemiological Statistics for Public Health), version 3.01 (http://www.openepi.com/Menu/OE_Menu.htm, accessed on 11 January 2024). Based on the above parameters, 28 patients per group had to be enrolled in the study. Accounting for a 15% dropout, it was planned to enroll at least 32 patients per group. Further details can be seen in Figure 1, which presents a Consort diagram.

### 2.5. Participants

All patients were recruited from those attending the Department of Dentistry and Maxillofacial Surgery at the “G.B. Rossi” University Hospital in Borgo Roma, Verona, between November 2023 and May 2024. All recruited patients underwent the extraction in the surgical rooms of the same department. The operators were specialist doctors or specialist residents in Maxillofacial Surgery and dentists training in oral surgery.

### 2.6. Randomization

The allocation of patients to the test and control groups was performed randomly according to a computer-generated sequence (https://www.openepi.com/Random/Random.htm, accessed on 11 January 2024) before the surgical extraction of the molars.

### 2.7. Surgical Procedures

In both groups, at the time of the third molar extraction (T0), demographic and anamnesis data were recorded, and the extracted tooth was classified based on the degree of inclusion and according to the Winter and Pell–Gregory classifications, based on radiographic examination (orthopantomography and/or CT). The degree of inclusion was classified as follows: tooth present in the arch, partially included tooth, mucosal inclusion, and bone inclusion. Periodontal indices were also recorded to evaluate the soft tissues, with measurements taken on the second lower molar adjacent to the tooth being extracted.

**Figure 2:**
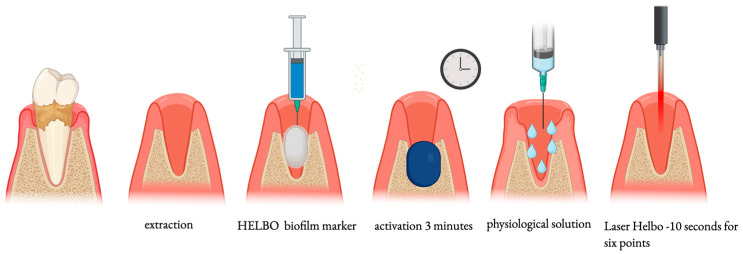
The steps of the antimicrobial photodynamic therapy (aPDT) protocol for the disinfection of the post-extraction socket.

After recording the periodontal indices and completing the clinical evaluation, the surgical procedure for the extraction of the mandibular third molar was performed, which included the local anesthesia (2% lidocaine and 1:100,000 epinephrine) of the inferior alveolar nerve and infiltrative anesthesia at the site of the incision. A trapezoidal flap was elevated, and the tooth was extracted. During the extraction, the need for osteotomy, odontotomy, and root separation was recorded. The test group received antimicrobial photodynamic therapy (aPDT) immediately before suturing. A diode laser with a wavelength of 660 nm was used (Helbo^®^ TheraLite Laser, Bredent, Germany). The operating protocol for the disinfection of the post-extraction socket provided by the manufacturer was used for this study. In brief, tooth extraction was carried out with caution. A careful but gentle curettage was applied to remove the granulation tissue in the apical and periodontal area of the socket. A strip of gauze was soaked with Helbo^®^ Biofilm Marker, and the socket was swabbed. This liquid is based on phenothiazine chloride and was provided in pre-packaged sterile syringes. The dye was applied to the entire area so the gauze strip would absorb the blood at the same time. The photosensitizer was left in the post-extraction socket for three minutes. This action time in the alveolus guaranteed the penetration of the coloring substance into the biofilm. Before irradiation with the Helbo^®^ TheraLite laser, excess photosensitizer was removed by rinsing with saline solution, and the remaining dye on the alveolar walls was irradiated with laser light. The laser was transmitted by means of sterile disposable optical fibers (Helbo^®^ 3D Pocket Probe, Bredent, Germany), which emit tridimensional light on the walls of the alveolus. Optical fiber was applied near the alveolar walls but without touching them. All alveolar walls were irradiated for ten seconds each (at the disto-lingual, lingual, mesio-lingual, mesio-buccal, buccal, and disto-buccal sites) for a total duration of 60 s. The timing of the liquid application and laser irradiation for each patient was controlled by the Helbo^®^ T-Controller device provided by the manufacturer. The remaining coloring substance on the socket walls absorbed the laser rays, which also prevented the spread of light. In cases of heavy bleeding from the socket, the blood was aspirated; in the meantime, the optical fiber was cleaned with a damp swab. After completing antimicrobial photodynamic therapy, the post-extraction socket was sutured with Vicryl 4.0 sutures (Figure 2). In the control group, once the tooth was removed, the post-extraction site was cleaned by curettage, washed with saline, and then sutured with a Vicryl 4.0 suture. The duration of the surgery, from the incision to the completion of the suturing, was recorded, as well as the degree of intraoperative bleeding. The bleeding was classified with a score from 0 to 2, where 0 indicates normal bleeding, 1 indicates moderate bleeding without the need for hemostasis, and 2 indicates excessive bleeding requiring hemostasis.

At the end of the surgical procedure, post-operative instructions were provided to the patients, and antibiotic therapy with analgesics was prescribed (Analgesics: Ibuprofen, 600 mg, 1 tablet every 12 h for 4 days; ketoprofen 100 mg, 1 tablet every 12 h for 4 days; or paracetamol 1000 mg tablets, 1 tablet every 12 h for 4 days). Post-surgical follow-ups were scheduled for 14 days (T1) and 3 months (T2) after the extraction for all patients.

### 2.8. Outcome Variables

Primary Outcome: The healing of the post-extraction socket was evaluated by assessing the PPD (probing pocket depth) at the adjacent molar.

Secondary Outcomes: Changes in the periodontal indices were evaluated at the second molar adjacent to the extraction site: BOP (bleeding on probing); the PI (plaque index); REC (gingival recession); CAL (clinical attachment loss); patient-reported pain levels; swelling; the number of painkillers taken; Landry’s healing index; and the evaluation of bone healing through intraoral radiographs. The following outcome variables were assessed at T0, T1 and T2 using a millimeter-marked manual periodontal probe (the double-ended probe P3N, HuFriedy Italy Srl, Milan, Italy):

- PPD: Measurements from the gingival margin to the base of the sulcus or pocket at six sites around the tooth using a manual periodontal probe.
- BOP: Recordings were made 30 s after probing from six sites around the tooth using a manual periodontal probe.
- PI: Recordings with a periodontal probe, assigning a value between 0 and 3 according to the criteria outlined by Silness and Löe in 1964 at six sites around the tooth.
- REC: Measurements from the cementoenamel junction to the free gingival margin using a manual periodontal probe at six sites around the tooth.
- CAL: The sum of PPD and REC, measured only on the buccal side (mesio-vestibular, vestibular, and disto-vestibular sites), as the lingual side is considered clinically irrelevant, considering that trapezoidal incisions involve the buccal side, with direct effects on the periodontal healing of this side.

The periodontal indices of PPD, BOP, PI, and REC were collected from three vestibular and three lingual sites (mesio-vestibular, vestibular, disto-vestibular, mesio-lingual, lingual, and disto-lingual sites) for each tooth at time T0, which was calculated and compared with the mean value of the three vestibular sites at times T1 and T2.

- The probing of the post-extraction site: The post-extraction socket was probed using a manual periodontal probe to assess healing.
- The presence of secretion: Any secretion from the post-extraction site was recorded.
- Landry’s healing index: The periodontal tissue healing index is based on tissue color, the tissue’s response to probing, and the evaluation of the incision margin. The score ranges from 1 to 5 as follows:

Code 1 (very poor healing): More than 50% of the gingiva is red in color, the presence of bleeding when manipulating the tissue, a non-epithelialized incision margin with loss of epithelium beyond the incision margin, and the presence of suppuration;

Code 2 (poor healing): More than 50% of the gingiva is red in color, the presence of bleeding when manipulating the tissue, the presence of granulation tissue, a non-epithelialized incision margin, and exposed connective tissue;

Code 3 (good healing): An amount of gingiva between 25% and 50%, the absence of bleeding when manipulating the tissue, the absence of granulation tissue, and lack of exposed connective tissue on the incision margin;

Code 4 (very good healing): Reddened gingiva levels less than 25%, no bleeding when handling the tissue, no granulation tissue, and no exposed connective tissue at the incisal edge;

Code 5 (excellent healing): Pink gingival tissue, no bleeding when handling, no granulation tissue, and no connective tissue at the incision edge.

- Bone filling: This was assessed through intraoral radiographs, comparing socket filling in mm of height between T0 and T2.
- Additionally, the following parameters reported by patients were recorded:
- Swelling: This was graded from 0 to 3, where 0 indicates no swelling, 1 indicates mild swelling, 2 indicates moderate swelling, and 3 indicates severe swelling.
- Number of painkillers taken: Patients were asked to report the number of painkillers taken from the day of the extraction to 14 days post-extraction.
- Pain: Patients reported their pain levels using the VAS scale at 14 days post-extraction.

The bone density index at the extraction site was also assessed through peri-apical X-rays at T2, which was attributed to a value from 0 to 2. A value of 0 corresponded to a low degree of bone density, with radiolucency in correspondence with the extracted dental element and marked radiopacity; a value of 1 corresponded to a medium density with slight radiopacity at the extracted third molar, compatible with the apposition of new immature bone and the persistence of marked radiopacity; a value of 2 corresponded to a high bone density with radiopacity in correspondence with the extracted element, compatible with new bone apposition.

### 2.9. Statistical Analysis

All the data were collected by the same clinician to avoid problems concerning inter-operator calibration and the reproducibility of the data. The measurement of outcomes was performed by another operator different from the surgeon who performed the extractions. Before the start of the study, this investigator was calibrated for adequate intra-examiner levels of accuracy and reproducibility in recording clinical, radiographic, and patient-reported parameters. Three clinical cases were utilized for this purpose: duplicate measurements were collected with an interval of 24 h between the first and second recordings. The intra-class correlation coefficients, used as a measure of intra-examiner reproducibility, had to be greater than 0.8.

Data were recorded in a Microsoft Excel database and checked for errors. Statistical analyses were performed using Stata v.13.0 (StataCorp, College Station, TX, USA). The Shapiro–Wilk test was used to assess the normality of the data. Continuous variables with a normal distribution were expressed as the mean ± standard deviation, while non-normally distributed variables were presented as medians with interquartile ranges (iqr). Qualitative data were reported as frequencies and percentages. Pearson’s chi-squared test was used to analyze the association between qualitative variables, and Fisher’s exact test was used when expected cell counts were less than five.

The comparison between the means of continuous variables at two different times was performed using the Wilcoxon matched-pairs signed-rank test. The comparison between the means of the two different groups was performed using the unpaired Wilcoxon rank-sum test. Bonferroni correction for multiple comparisons was applied. A *p*-value of less than 0.05 was considered statistically significant.

## 3. Results

### 3.1. Patients’ Demographics

In total, 65 patients (22 males and 43 females) were included and randomly divided into the test and control groups composed of 32 and 33 patients, respectively. The mean age of the patients was 26.23 ± 6.07 years (range 14 to 39 years). In terms of health status, 60 patients were classified as ASA I, and 5 patients were classified as ASA II. Seventeen patients had smoking habits. Preoperative panoramic radiographs and computerized tomography were performed in 43 (66.15%) and 46 (70.77%) cases (24 cases had both), respectively. Regarding the degree of impaction, 10 teeth were fully erupted, 29 were partially impacted, 9 were mucosally impacted, and 17 were fully impacted. The sample was further classified according to the Winter and Pell–Gregory classification: 32 teeth were vertical, 8 were horizontal, 20 were mesio-angulated, and 5 were disto-angulated. Table 1 summarizes patients’ demographics and the intra-surgical parameters per group.

### 3.2. Post-Operative Assessment

At the 14-day follow-up, one patient from the control group was lost to follow-up. During the three-month follow-up, two patients from each group were lost to follow-up, resulting in a total of five dropouts (three from the control group and two from the test group). The statistical analysis was based on data from 60 patients. The mean values of PPD, BOP, PI, REC, and CAL at T0, T1, and T2, as well as the differences between T0 and T1 and between T1 and T2, can be seen in detail in Table 2 with *p*-values relative to intra-group and inter-group comparisons for each outcome variable.

Regarding the soft tissue assessment, each periodontal index was evaluated considering the average value of the three vestibular sites at T0, T1, and T2 for both the overall sample and for each group separately.

A significant increase in PPD (*p* < 0.05) was observed between T0 and T1 in both groups. The inter-group comparison of PPD variations was not significant between groups (*p* = 0.28). In the T1–T2 interval, a significant decrease (*p* < 0.05) in PPD was observed for each group despite no significant difference being found between groups (*p* = 0.2).

For BOP, an inter-group comparison for the variations in the T0–T1 interval was significant (*p* = 0.002). For the interval T1–T2, a non-significant decrease was recorded in both groups, with no significant inter-group difference.

For PI, a significant increase between T0 and T1 was observed in the test (*p* = 0.001) but not in the control group. For the interval T1–T2, the test group revealed a significant decrease instead (*p* = 0.02); furthermore, a significant difference between groups regarding variations was found (*p* = 0.002).

For REC, the control group was stable throughout the study, and the test group improved between T0 and T1 (*p* = 0.001) and remained steady thereafter. There was no significant inter-group difference in variations between T1 and T2 (*p* = 0.3)

CAL at T0 was significantly worse (*p* = 0.002) in the test group compared to the control group. There was a significant inter-group difference (*p* = 0.002) between the T0–T1 variations, with a non-significant variation between T1 and T2 (*p* = 0.11). The intra-group decrease from T1 to T2 was significant (*p* < 0.05) for both groups.

Post-operative swelling, pain (VAS index), the quantity of painkillers taken, alveolar probing, and Landry’s healing index are listed in detail in Table 3.

As regards the parameters of swelling and pain, these parameters were significantly lower (*p* < 0.05) in the test group compared to the control group. As regards the quantity of painkillers taken in the post-intervention follow-up, this value was, on average, lower in the test group compared to the control group, even if the difference was not statistically significant (*p* = 0.22). For the comparison between the test group and the control group on Landry’s healing index, effective healing was found for both groups despite no significant differences (*p* = 0.75). Secretion from the socket and suture loss at the T1 follow-up were recorded in one and seven patients, respectively.

Bone healing results at post-extraction sites assessed with intraoral X-rays at T2 are listed in Table 4.

At the three-month follow-up (T2), bone healing was assessed through intraoral X-rays: no statistically significant differences were found between the groups, with both groups presenting medium bone density (*p* = 0.56).

## 4. Discussion

The surgical extraction of impacted mandibular third molars is one of the most common procedures in oral and maxillofacial surgery [13,14,15,16,17]. This study aimed to observe the effects of aPDT on the healing of the post-extraction socket of the mandibular third molar in a short-term follow-up study. According to the results, antimicrobial photodynamic applications as a form of adjunctive therapy after the surgical removal of inferior third molars can be considered beneficial for a better post-operative course in promoting wound healing with lower levels of discomfort when compared to the control group. The aPDT seemed to be associated with less swelling and lower pain, and the patients used fewer painkillers, even if the difference with the control group was not statistically significant.

In this study, to investigate the influence of aPDT on the healing of soft tissues, the Landry index and the periodontal indices PPD, BOP, PI, REC, and CAL were used [18,19,20]. In more detail, for the interval T1–T2, a significant decrease (*p* < 0.05) of PPD was observed for each group despite no significant difference being found between groups (*p* = 0.2). For BOP, as regards the interval T1–T2, a non-significant decrease was recorded in both groups, with no significant inter-group difference. For PI, for the interval T1–T2, the test group revealed a significant decrease instead (*p* = 0.02); furthermore, a significant difference between the groups was found regarding variations (*p* = 0.002). It is interesting to observe that aPDT is associated with a reduction in the plaque index, which seems to support the antimicrobial effect of the therapy in decreasing the bacterial colonization of the post-extraction site, with a possible additional benefit at the level of the adjacent second molar. For REC, the control group was stable throughout the study, and the test group improved between T0 and T1 (*p* = 0.001) and remained steady thereafter. There was no significant inter-group difference in variations between T1 and T2 (*p* = 0.3). Regarding CAL, there was a significant inter-group difference (*p* = 0.002) between the T0–T1 variations, with a non-significant variation between T1 and T2 (*p* = 0.11). The intra-group decrease from T1 to T2 was significant (*p* < 0.05) for both groups. Even if the absence of a measurement of CAL on the lingual side could represent a potential bias in the final outcomes, this can be considered clinically irrelevant, as trapezoidal incisions involved the buccal side, with direct effects on the periodontal healing of this side. Moreover, CAL at T0 was significantly worse (*p* = 0.002) in the test group compared to the control group: this issue represents a possible limitation for the interpretation of the results, as baseline values can undermine post-treatment comparisons since differences may be determined based on the initial status, not on the effects of treatment. Based on this, studies with a larger sample size are needed to corroborate the effective improvement of CAL following aPDT in the test group.

Concerning the VAS index, this parameter was significantly lower (*p* = 0.04) in the test group compared to the control group; however, this outcome can be considered clinically irrelevant (1.5 vs. 0.7) in terms of absolute difference. Considering the Landry index, the use of aPDT did not appear to significantly influence the healing of the soft tissues of the post-extraction site, with similar and better healing results fourteen days after extraction for both groups. To understand if aPDT had an effect on the healing of bone tissue, all patients underwent intraoral radiography of the post-extraction site at three months. As a result, the use of aPDT did not significantly affect bone healing, which was incomplete for all the cases that were observed. This is probably because the three-month interval is too short to evaluate complete bone remodeling [21,22,23,24]. Various reports in the literature show more advanced bone healing following wisdom tooth extractions in combination with or without aPDT when extending the radiographic follow-up period to six months [25,26,27,28,29,30].

Currently, there are few studies in the literature regarding the use of aPDT associated with the extraction of mandibular third molars [7,11,28,29,30,31,32,33,34]. Moreover, the majority of these study the effects of antimicrobial photodynamic therapy on pain, swelling, trismus, and halitosis. There are no studies in the literature that evaluate periodontal indices following the surgical avulsion of the lower third molar in association with aPDT. As regards the association between aPDT and the degree of swelling and pain reported by the patient in the post-surgical period, the results obtained in the present study are in line with those mentioned in the literature for low-level laser therapy [34,35,36]. In a study by Batinjan et al. [34], it was highlighted that the use of aPDT on the post-extraction socket resulted in less discomfort for the patient in the post-operative period in terms of swelling, perceived pain intensity, and halitosis compared to the group subjected to LLLT (low-level laser therapy) and the control group. This difference was statistically significant three days after extraction, while it was comparable to that of the group subjected to LLLT at the check-up on the seventh and fourteenth days, but still lower than the control group, which had the worst outcome in the post-operative period [35]. Although there are no studies relating to the use of antimicrobial photodynamic therapy checked using periodontal indices, it is possible to compare the variation in the parameters of the control group with the data present in the literature [35,36,37,38]. Studies conducted on periodontal indices following the extraction of impacted mandibular third molars in the literature do not present consistent results. In particular, in a study by Faria et al. [36], PPD, BOP, PI, REC, CAL, and GI (gingival index) were evaluated in five sites at the second molar adjacent to the extracted element (buccal, disto-buccal, central to the distal surface, disto-lingual, and lingual sites) before extraction, and at three months, six months, and twelve months after extraction in a sample of 25 patients. The results obtained showed a general decrease in all periodontal indices from the pre-extraction check-up to the three-month check-up, with a significant improvement in the periodontal health of the second molar adjacent to the post-extraction site, while the differences in the subsequent intervals were not statistically significant. This data allows us to hypothesize that the periodontal healing of the lower second molar is achieved at three months. Another study reports that there are no significant differences between the preoperative measurements of periodontal indices and the measurements taken at the post-operative check-up at six and twelve months, with values that are almost unchanged [37]. The results obtained in the present study showed better outcomes in the control group (except for the PI values) at the three-month check-up compared to the time of extraction. This could be due to incomplete healing, which leads to the accumulation or stagnation of food in the area affected by the operation, with consequent difficulty in oral hygiene maneuvers and an increase in the values of the plaque index. According to the results of this present study, aPDT seemed to improve periodontal indices; however, at T2, most indices did not show significant between-group differences. The benefit thus seemed transient or confounded by baseline differences (e.g., CAL). As such, the improvement of the clinical attachment can be considered transitory in the sense that once it reaches its maximum level, it stabilizes and remains constant. Furthermore, for bone healing, the efficacy was greater in the initial phases following extraction; the immediate post-operative period showed improvement, which was consistent with the short follow-up. The suggested benefit is referable only to the results that were statistically significant in the immediate post-operative period. In the long term, this difference was not evident.

Additional advantages of aPDT can be a reduction in costs, which might be linked to taking fewer medications, such as fewer painkillers or antibiotics with antimicrobial resistance benefits, but these statements are not appropriate at this stage. Currently, there is a lack of clinical reports in the literature to confirm that aPDT applications can reduce antibiotic usage, and further investigation should be conducted to assess these possible advantages.

Major limitations of this study consist of a limited number of subjects, the overlap of any associated antibiotic therapy, the analysis of CAL from the buccal side only, and the short-term follow-up period of 3 months, which can be misleading for the evaluation of bone healing; however, this study was planned from the beginning as a pilot study, and there is ongoing research in our department on this topic. Further investigations are needed to confirm the results obtained, using larger sample sizes with at least 6 months of follow-up and the evaluation of bone density through Housfield units, the evaluation of patient satisfaction using quality of life questionnaires with comparison to other popular adjunctive therapies such as low-level laser treatment or autologous platelet concentrates. A possible future aim could be the analysis of the antimicrobial effect of aPDT therapy as a replacement for the use of antibiotic therapy to determine if aPDT is an option to address medical problems related to antibiotic resistance. However, further scientific research is needed to clarify this question.

## 5. Conclusions

According to the results of this study, a significant increase in PPD was observed between T0 and T1 in both groups. In the interval T1–T2, a significant decrease in PPD was observed for each group. No significant difference was found for PPD between the groups at any time interval. For BOP, an inter-group comparison for the variations in interval T0–T1 was significant, while interval T1–T2 showed a non-significant decrease for both groups with no significant inter-group difference. For PI, a significant increase between T0 and T1 was observed during the test, but this was not observed in the control group. For the interval T1–T2, the test group had a significant decrease with a significant difference between groups. REC outcomes were mostly stable, and the test group showed some improvements between T0 and T1. There was no significant inter-group difference in variations. In the test group, CAL at T0 was significantly worse compared to the control group. There was a significant inter-group difference at T0–T1, with a non-significant variation between T1 and T2. The intra-group decrease from T1 to T2 was significant for both groups.

To conclude, this study demonstrates that antimicrobial photodynamic therapy (aPDT) can have positive effects on the healing of post-extraction sockets after the removal of impacted mandibular third molars. The use of aPDT in this study significantly reduced post-operative swelling and pain in 14 days, with additional benefits for periodontal health, such as a reduction in the plaque index and improved clinical attachment levels at three months. Although the effect of aPDT on bone healing was not significant within the short observation period, further studies with longer follow-up durations are needed to fully assess the potential of aPDT in promoting bone healing after tooth extraction.

## Figures and Tables

**Table 1:** Pre-surgical and intra-surgical information and between-group comparisons.

Variables		Control	Test	*p*-Value
**Gender**	Male	12	10	0.66
	Female	21	22	
**Age (years: mean ± SD)**		26.27 ± 5.95	26.18 ± 6.28	0.76
**General health Status**	ASA 1	30	30	0.51
	ASA 2	3	2	
**Smoking habit**	No	23	25	0.44
	Yes	10	7	
**Tooth element**	38	13	21	0.03 *
	48	20	11	
**Tooth inclusion**	Fully Erupted	2	8	0.16
	Partially Impacted	15	14	
	Mucosal Impaction	6	3	
	Bony Impaction	10	7	
**Winter Classification**	Vertical	15	17	0.8
	Horizontal	5	3	
	Mesio-Angulated	11	9	
	Disto-Angulated	2	3	
**Pell and Gregory Classification**	Code IA	9	15	0.14
	Code IB	13	6	
	Code IC	6	7	
	Code IIA	1	3	
	Code IIB	4	1	
**Ostectomy**	No	1	4	0.16
	Yes	32	28	
**Odontotomy**	No	13	15	0.54
	Yes	20	17	
**Root Separation**	No	23	25	0.44
	Yes	10	7	
**Intra-Operative Bleeding**	Normal	26	18	0.17
	Moderate	4	8	
	Significant	3	6	
**Post-Operative Suppuration**	No	31	32	0.5
	Yes	1	0	
**Post-Operative Suturing**	No	29	28	0.5
	Yes	3	4	
**Operating Time, minutes (mean ± SD)**		30.6 ± 17.42	34.43 ± 16.63	0.33

*: statistically significant; SD: standard deviation; ASA: American Society of Anesthesiologists.

**Table 2:** Evaluation and comparison of mean values of probing pocket depth (PPD), bleeding on probing (BOP), plaque index (PI), gingival recession (REC), and clinical attachment loss (CAL) before surgery (T0) after 14 days (T1), and after 3 months (T2). Values for all parameters are presented as median (iqr) [min–max] at each time interval.

		T0	T1	∆(T0–T1)	*p*-Value (Intra-Group Comparison)	T2	∆(T1–T2)	*p*-Value (Intra-Group Comparison)
**PPD**	Control group	2.49 (0.59) [1–3.33]	3.68 (1.05) [2–6.66]	1.08 (1.24) [(−2.33)–4.66]	0.001 *	1.81 (0.84) [1–4.33]	−1.92 (1.42) [(−5)–0.66]	0.002 *
	Test group	2.15 (0.76) [2–3.66]	2.95 (1.32) [1–7]	0.8 (1.61) [(−2)–6]	0.009 *	1.71 (0.57) [1–3]	−1.35 (1.54) [(−6)–1]	0.002 *
	*p* value(inter-group comparison)	0.04 *	0.002 *	0.28		0.99	0.2	
**BOP**	Control group	0.02 (0.08) [0–0.33]	0.12 (0.26) [0–1]	0.1 (0.24) [(−0.33)–1]	0.02 *	0.1 (0.28) [0–1]	−0.03 (0.37) [(−1)–1]	0.42
	Test group	0.0 (0.00) [0–0]	0.2 (0.37) [0–1]	0.2 (0.37) [0–1]	0.25	0.06 (0.18) [0–0.66]	−0.14 (0.44) [(−1)–0.66]	0.11
	*p* value (inter-group comparison)	0.16	0.12	0.002 *		0.92	0.48	
**PI**	Control group	0.25 (0.55) [0–2]	0.07 (0.25) [0–1]	−0.18 (0.58) [(−2)–1]	0.13	0.51 (0.72) [0–3]	0.39 (0.7) [(−1)–3]	0.006 *
	Test group	0.12 (0.27) [0–1]	0.78 (0.94) [0–3]	0.65 (0.97) [(−1)–3]	0.001 *	0.50 (0.82) [0–3]	−0.31 (0.96) [(−2)–3]	0.02 *
	p value (inter-group comparison)	0.82	0.002 *	0.002 *		0.64	0.002 *	
**REC**	Control group	0.00 (0.00) [0–0]	0.00 (0.00) [0–0]	0.00 (0.00) [0–0]	/	0.00 (0.00) [0–0]	0.00 (0.00) [0–0]	/
	Test group	1.12 (1.09) [0–3]	0.2 (0.11) [0–0.66]	−1.1 (1.08) [(−3)–0]	0.001 *	0.00 (0.00) [0–0]	−0.02 (0.11) [(−0.66)–0]	0.31
	*p* value (inter-group comparison)	0.002 *	0.31	0.002*		/	0.30	
**CAL**	Control group	2.49 (0.59) [1–3.33]	3.68 (1.05) [2–6.66]	1.08 (1.24) [(−2.33)–4.66]	0.001 *	1.81 (0.84) [1–4.33]	−1.92 (1.42) [(−5)–0.66]	0.002 *
	Test group	3.28 (1.58) [1–6]	2.97 (1.33) [1–7]	−0.3 (2.06) [(−4)–6]	0.25	1.71 (0.57) [1–3]	−1.37 (1.54) [(−6)–1]	0.006 *
	*p* value (inter-group comparison)	0.002 *	0.31	0.002 *		0.99	0.11	

PPD = probing pocket depth; BOP = bleeding on probing; PI = plaque index; REC = gingival recession; CAL = clinical attachment loss; T0 = before surgery; T1 = post-operative 14 days; T2 = post-operative 3 months. *: statistically significant; Bonferroni correction was applied.

**Table 3:** Post-operative swelling, pain (VAS index), quantity of painkillers taken, alveolar probing, and Landry’s healing index. Values for all parameters are presented as n (%) [C.I.] or mean ± SD [min–max].

Variables at T1	Values	Control	Test	*p*-Value
Swelling	None	11 (34.38) [0.42–0.66]	24 (75) [0.35–0.76]	0.002 *
	Minor	17 (53.14) [0.27–0.51]	8 (25) [0.04–0.23]	
	Moderate	4 (12.5) [0.44–0.56]	0 (0) [0.16–0.47]	
VAS index		1.5 ± 1.84 [0–6]	0.7 ± 1.21 [0–4]	0.04 *
Quantity of painkillers (no. of tablets)		12.87 ± 8.36 [1–30]	10 ± 6.48 [2–24]	0.22
Landry’s healing index	Code 2	2 (6.25) [0.03–0.17]	3 (9.38) [0.26–0.71]	0.75
	Code 3	12 (27.5) [0.26–0.5]	12 (37.5) [0.05–0.19]	
	Code 4	17 (53.13) [0.4–0.65]	17 (53.13) [0.14–0.37]	
	Code 5	1 (3.13) [0.02–0.1]	0 (0) [0.04–0.1]	

*: statistically significant.

**Table 4:** Bone healing results at post-extraction sites assessed with intraoral X-rays at T2. Values for all parameters are presented as n (%) [C.I.].

Bone Density	Control	Test	*p*-Value
Low (code 0)	5 (16.67) [0.17–0.41]	7 (23.33) [0.2–0.67]	0.56
Medium (code 1)	15 (50.00) [0.04–0.25]	11 (36.67) [0.5–0.19]	
High (code 2)	10 (33.33) [0.08–0.43]	12 (40.00) [0.25–0.51]	

## Data Availability

The original contributions presented in this study are included in the article. Further inquiries can be directed to the corresponding author.

## Abbreviations

The following abbreviations are used in this manuscript:

aPDT	Antimicrobial photodynamic therapy
PI	Plaque index
REC	Gingival recession
CAL	Clinical attachment loss
PPD	Probing pocket depth
BOP	Bleeding on probing
LLLT	Low-level laser therapy
